# Organizational and Nursing Management Factors Associated With Guideline‐Discordant Medical Radiation Protective Apparel Disinfection: A Multicenter Interpretable Machine Learning Study

**DOI:** 10.1155/jonm/2036458

**Published:** 2026-11-08

**Authors:** Xing Xin, Suwen Li, Chenxin Ye, Luyin Cheng, Yuting Wu, Gendi Lu

**Affiliations:** ^1^ Department of Nursing Shanghai Guangming Hospital of Traditional Chinese Medicine Affiliated to Shanghai University of Traditional Chinese Medicine Shanghai China; ^2^ Clinical Research Center Shuguang Hospital Affiliated to Shanghai University of Traditional Chinese Medicine Shanghai China shutcm.edu.cn

**Keywords:** cross-sectional study, guideline adherence, implementation, machine learning, nursing education, radiation protection

## Abstract

**Background:**

Effective management of Medical Radiation Protective Apparel (MRPA) is a global implementation challenge. In China, this is complicated by discordant guidance regarding MRPA disinfection, with some domestic specialty recommendations differing from manufacturer‐aligned material‐care guidance.

**Aim:**

To identify organizational and nursing management factors associated with guideline‐discordant MRPA disinfection using an interpretable machine learning approach and to characterize their joint predictive patterns.

**Participants and settings:**

A total of 123 public hospitals in two developed regions of eastern China (Shanghai and Zhejiang Province, China), including Tertiary A, Tertiary B, and Secondary A institutions.

**Methods:**

In this cross‐sectional study, a self‐designed structured survey assessed MRPA management practices, with Critical Noncompliance (CNP)—a study‐defined endpoint referring to reported use of alcohol‐ or chlorine‐based disinfectants—as the primary outcome. This classification reflected discordance with manufacturer‐aligned material‐care recommendations rather than a formal regulatory designation. Data were analyzed using descriptive statistics and interpretable machine‐learning models, with random forest selected as the final model and SHAP used for model interpretation.

**Results:**

Guideline‐discordant practices were common: alcohol‐ and chlorine‐based disinfection was reported by 63.4% and 65.9% of institutions, and 67.5% used manual or partly manual traceability. Nursing personnel held 81.7% of management roles. In the independent test set, the random forest model achieved an AUROC of 0.849 (95% CI, 0.558–0.922). Random forest and SHAP identified Tertiary Grade A status, critical‐area traceability, designated personnel, disinfection‐personnel training, housekeeping/environmental‐services execution, and third‐party laundering as prominent contributors to CNP prediction.

**Conclusions:**

Guideline‐discordant MRPA disinfection was associated with multiple organizational and workflow characteristics rather than a single professional role or management feature. The random forest–SHAP patterns are hypothesis‐generating and identify organizational domains for prospective validation, particularly those involving guidance, training, traceability, and workflow design.

**Implication for Nursing Management:**

Nursing roles should be considered within the broader organizational systems in which responsibility, training, traceability, and workflow are structured. Future implementation studies should evaluate whether coordinated alignment of these components with evidence‐based MRPA care improves guideline adherence, particularly in higher‐tier hospitals.

## 1. Introduction

Medical Radiation Protective Apparel (MRPA), including lead aprons and thyroid shields, is essential personal protective equipment in ionizing radiation environments, intended to reduce occupational and patient radiation exposure risk [1]. A common misconception in practice treats MRPA protective performance as a fixed attribute determined solely by procurement quality, overlooking that its effectiveness is dynamic and dependent on standardized management across its full lifecycle—procurement, use, storage, decontamination, and retirement. Suboptimal management can degrade shielding performance, increase pathogen transmission risk via surface contamination, and accelerate material aging, thereby shortening service life. Clinical implementation of systematic MRPA management remains inconsistent, and failures are frequent. Poor handling not only diminishes protection but can compromise sterile fields and exacerbate contamination and material degradation [2]. Despite clear recommendations in manufacturer manuals, international standards, and recent studies regarding appropriate configuration and cleaning of protective equipment, substantial gaps persist between guidelines and practice. For instance, a multicenter study in Togo found 62.79% of lead aprons noncompliant with standards: 46.51% were improperly stored, 16.28% folded, and only 32.56% correctly hung. A study in Nigeria similarly reported nearly half of protective gear suffered severe damage from improper use or storage [3, 4].

MRPA contamination has attracted increasing concern. These items are frequently used in ostensibly sterile environments such as operating and interventional suites and are repeatedly exposed to healthcare workers and patient surroundings. Inadequate cleaning frequency or improper methods facilitate colonization by multidrug‐resistant organisms, particularly biofilms with high tolerance to disinfectants [5]. The U.S. National Institutes of Health (NIH) notes biofilms contribute to 65%–80% of chronic infections, creating a patient safety risk when present on MRPA [6]. According to the 2025 guidelines from the Association of perioperative Registered Nurses (AORN), contamination rates of lead apparel range from 62.5% to 100%, while many institutions demonstrate deficiencies or lapses in cleaning and disinfection execution. Studies report microbial loads often exceed acceptable facility thresholds [7, 8]; an Irish investigation found 100% of inspected thyroid shields were contaminated [9].

Existing literature has largely concentrated on healthcare workers’ knowledge, attitudes, and behaviors regarding radiation protection or on device performance and dose monitoring, with comparatively few studies focusing specifically on the full‐lifecycle management of MRPA. A 4‐year evaluation of 1011 MRPA items reported 47.3% had tears and 31% required decommissioning; among 287 newly procured items, 6.0% failed within the first year, of which 88.2% were rendered unusable [2]. Other work highlights widespread gaps in radiation protection awareness among clinical staff—for example, a Japanese survey identified deficiencies in both equipment availability and educational support in endoscopy units, leaving MRPA cleaning and management underexamined [10]. Although bodies such as The Joint Commission and AORN have articulated requirements for cleaning and maintenance, unclear responsibility assignment has impeded consistent implementation. A U.S. survey found that while 78% of respondents acknowledged contamination risk, 48% reported never having cleaned protective apparel and only 15% indicated their institution had clear policies. Such findings suggest that gaps in MRPA management may involve not only individual knowledge or equipment availability, but also how responsibility for routine implementation is assigned and organized. Preliminary evidence further suggests that nurses constitute a substantial proportion of the personnel involved in the use and cleaning of protective apparel [11].

This concentration of nursing involvement shifts the question from whether nurses participate in MRPA management to how that participation is organized and supported. Evidence from nursing guideline implementation indicates that dissemination alone is insufficient to achieve consistent uptake in practice; implementation commonly involves multiple strategies, including education, local opinion leaders, audit and feedback, and context‐specific adaptation [12]. From a nursing‐management perspective, a metasynthesis of nurse managers’ experiences identified teamwork, organizational structures, and leadership as key elements facilitating evidence implementation. Nurse managers contributed through activities such as policy and protocol development, staff communication, audit and feedback, and professional development [13]. Together, these findings provide a rationale for examining nursing‐related management characteristics alongside broader organizational and workflow factors in MRPA guideline implementation.

In China, MRPA cleaning and disinfection are situated within a broader infection‐control framework that emphasizes compatibility with manufacturers’ instructions. For example, WS 310.2‐2016 requires cleaning of precision devices to follow manufacturers’ instructions or guidance manuals [14]. This manufacturer‐aligned principle is consistent with MRPA care recommendations from AORN and major manufacturers, which caution against cleaning agents that may damage protective materials [15]. Experimental studies have likewise shown that repeated wiping with alcohol‐ or chlorine‐containing products can produce measurable material degradation [16].

At the same time, recent domestic specialty guidance has endorsed different approaches for MRPA care. The 2023 report “Best Evidence for Proper Maintenance of Radiation Protective Equipment by Interventional Practitioners” assigns alcohol‐ and chlorine‐based disinfection an A‐level recommendation [17], while the 2024 “Expert Consensus on the Application and Management of Lead Radiation Protective Devices” also lists these agents as routine disinfection options [18]. This divergence between manufacturer‐aligned material‐care recommendations and domestic specialty guidance creates a clinically relevant implementation dilemma for institutions attempting to standardize MRPA practice.

This apparent “standard‐practice gap” may be understood not simply as a failure of knowledge translation but as a form of local adaptation to competing operational demands [19]. Such adaptations, often described as “workarounds” in complex healthcare systems, may emerge when formal recommendations are difficult to reconcile with local practice conditions [19, 20]. Similar implementation challenges have been documented in other complex equipment domains, including flexible endoscope reprocessing, where technically demanding workflows can complicate consistent adherence to recommended procedures [21].

These observations suggest that guideline‐discordant MRPA disinfection may reflect an organizational configuration rather than a single management feature. Governance, staffing, training, traceability, executor allocation, and quality‐control characteristics may coexist in different combinations across institutions, and their predictive relevance may not be adequately summarized by considering each factor in isolation. We therefore conducted a multicenter cross‐sectional study in public hospitals in Shanghai and Zhejiang Province and compared interpretable classification approaches to examine these organizational patterns. Random forest was used to explore nonlinear and potentially interacting predictive relationships, with SHapley Additive exPlanations (SHAP) applied to characterize feature contributions to model predictions, and LASSO logistic regression included as a linear comparator. The study aimed to identify organizational and nursing‐management characteristics associated with guideline‐discordant MRPA disinfection and to define patterns for prospective validation.

## 2. Methods

### 2.1. Study Design

This quantitative, descriptive cross‐sectional study collected data at a single time point to systematically assess MRPA management, institutional policies, and operational practices in public hospitals in Shanghai and Zhejiang Province. These two regions are core components of the Yangtze River Delta, where healthcare resource supply has increased substantially over the past decade, although important intercity and intraregional differences remain [22]. They were therefore selected to examine whether an MRPA guideline–practice gap remained evident within a comparatively well‐resourced eastern Chinese healthcare context. Eligible hospitals were public institutions at Secondary Grade A level or above that used MRPA in clinical settings involving ionizing radiation.

### 2.2. Research Tools

#### 2.2.1. A Survey on the Management, Use, and Decontamination of MRPA

Data were collected using a structured questionnaire developed by the research team—A Comprehensive Survey on the Management, Use, and Decontamination of MRPA in Healthcare Facilities (Supporting File 1)—based on the latest guidelines from the AORN [15] and U.S. Centers for Disease Control and Prevention (CDC) recommendations for noncritical equipment cleaning [23].

Content validity was assessed by a panel of five experts in radiation safety, infection control, and nursing management. Item‐level content validity indices (I‐CVI) ranged from 0.81 to 0.90, with a scale‐level index (S‐CVI) of 0.86, indicating high content validity. A pilot study evaluated test–retest reliability among 20 eligible managers, surveyed twice at a 3‐week interval. Core scale scores demonstrated strong stability (ICC = 0.85, 95% CI [0.70, 0.93]), and Cohen’s Kappa coefficients for key categorical variables exceeded 0.72.

The final questionnaire comprised 48 items covering seven domains: (1) institutional and respondent demographics; (2) governance and management structure; (3) staffing and training; (4) cleaning, disinfection, and storage procedures; (5) policies and protocols; (6) quality control and environmental monitoring; and (7) department‐specific operational pathways. Definitions and coding logic for all predictor variables are provided in Supporting File 2.

#### 2.2.2. Rationale: The Evidence‐Practice Gap in MRPA Management

To examine factors associated with guideline‐discordant disinfection practices, we defined a proxy endpoint—critical noncompliance (CNP), referring specifically to the reported use of alcohol‐ or chlorine‐based disinfectants for MRPA cleaning, irrespective of concentration or frequency. This operational definition was informed by three complementary considerations. First, national infection‐control guidance: WS 310.2‐2016 establishes the broader principle that cleaning of precision medical devices should follow manufacturers’ instructions or guidance manuals [14]. Second, MRPA care recommendations: AORN guidance and manufacturer‐aligned recommendations emphasize appropriate maintenance and material‐compatible cleaning practices for radiation protective equipment [15]. Third, empirical material safety: repeated exposure to alcohol‐ or hypochlorite‐based agents has been associated with physical degradation of the outer covering and localized mechanical damage to protective garments, despite no demonstrated reduction in radioprotective properties in the experimental study [16].

Accordingly, CNP was used as a study‐defined operational marker of disinfection practice that was discordant with manufacturer‐aligned material‐care recommendations. It was not intended as a formal determination of regulatory violation or as a comprehensive measure of overall MRPA management quality.

CNP was selected over a composite “best practice score” for methodological reasons. A composite index would conflate heterogeneous management domains (e.g., inspection practices and disinfection choice), thereby diluting analytical focus. CNP, as a discrete guideline‐discordant disinfection practice, enabled precise identification of predictive factors influencing this specific decision. The machine learning models in this study were designed to predict the occurrence of this risk behavior, not overall management quality, allowing us to examine organizational characteristics associated with this guideline‐discordant technical pathway.

### 2.3. Data Collection and Sampling Strategy

Data were collected online between June and August 2024 using a convenience sampling approach. Hospitals meeting the inclusion criteria in Shanghai and Zhejiang Province were recruited through the research team’s academic and clinical networks. Because recruitment was based on convenience sampling through these networks rather than a probability‐based regional sampling frame, the participating hospitals should not be considered statistically representative of all eligible public hospitals in Shanghai or Zhejiang Province. A purposive targeting strategy was employed to invite key informants—such as radiation safety officers, infection control leads, and head nurses—who held direct responsibility and sufficient knowledge of MRPA management.

To maximize the response rate, three measures were implemented: (1) invitations were distributed via established collaboration networks; (2) the questionnaire’s introduction emphasized the study’s academic purpose and potential practical value; and (3) polite reminders were sent via email or phone to nonrespondents. Of 130 questionnaires distributed, 123 valid responses were obtained (94.6% response rate).

Data quality was strictly controlled during the collection phase. Since the electronic survey platform required all mandatory items to be completed before submission, only complete questionnaires were captured. Consequently, a complete case analysis was performed on the 123 valid responses, with no missing data present in the final dataset.

The study adhered to the STROBE guidelines [24]. All participants reviewed the study description and provided electronic informed consent before participating. Data were anonymized, kept strictly confidential, and used solely for academic analysis and healthcare quality improvement, with no identifiable personal or institutional information retained.

### 2.4. Statistical Analysis

Statistical analyses were conducted using SPSS 26.0 and RStudio 4.5.1. All hypothesis tests were two‐tailed, with *p* < 0.05 considered statistically significant. Descriptive statistics (frequency, proportion, mean/median, and standard deviation/interquartile range) were used to summarize institutional characteristics and management practices. Relationships between variables were analyzed using Pearson’s chi‐square test (*χ* ^2^) and Fisher’s exact test. For multiple response data, multiple response cross‐tabulation was performed, and Bonferroni correction was applied to assess differences in the distribution of management practices across groups.

To explore organizational factors associated with CNP, three classification models were developed and compared: Random forest [25], XGBoost [26], and LASSO logistic regression [27]. The optimal model was selected based on predictive performance in an independent test set. Data were randomly split into training and independent test sets in a 7:3 ratio, with 10‐fold cross‐validation on the training set for hyperparameter tuning.

The random forest model used 500 trees (ntree = 500) after examination of out‐of‐bag error convergence, while mtry was tuned by 10‐fold cross‐validation over candidate values ranging from 2 to the smaller of 20 or the number of available predictors. XGBoost was tuned using the caret xgbTree implementation with a model‐generated tuning grid (tuneLength = 5), with AUROC used to select the optimal configuration. For LASSO logistic regression, alpha was fixed at 1 and lambda was tuned over values from 0.001 to 0.100 in increments of 0.005. For all three models, hyperparameter tuning was performed within the training data using 10‐fold cross‐validation with SMOTE applied only within the resampling process.

Random forest—a tree‐based ensemble algorithm well suited to high‐dimensional, nonlinear relationships [25]—was chosen for final analysis. To avoid information leakage, all variables directly linked to the endpoint or downstream of it (e.g., disinfection method, Q24) were excluded from the feature set. The final model included 22 binary predictors reflecting hospital characteristics, management and staffing, processes and equipment, and quality control measures. In both modeling and SHAP interpretability analyses, “presence of CNP” was defined as the positive class. Variable importance was assessed using permutation‐based importance and SHAP values, with results visualized accordingly.

Given the modest sample size (*N* = 123) and the imbalance between CNP and non‐CNP cases, we implemented a validation framework to improve model robustness and reduce the risk of overfitting. The dataset was first split into training and independent test sets in a 7:3 ratio. In the training set, there were 70 CNP cases (80.5%) and 17 non‐CNP cases (19.5%) before resampling. SMOTE was applied only within the training resampling process during 10‐fold cross‐validation, where the minority class was synthetically oversampled to achieve an approximately 1:1 class balance (50% CNP vs. 50% non‐CNP). The independent test set was not subjected to SMOTE and retained its original distribution of 29 CNP cases and 7 non‐CNP cases. Hyperparameters for all models (random forest, XGBoost, and LASSO logistic regression) were tuned using 10‐fold cross‐validation. To quantify statistical uncertainty, we calculated 95% confidence intervals (CIs) for the area under the curve (AUC) using bootstrap resampling with 2000 iterations. This framework was used to assess the robustness of model performance and reduce the likelihood that the findings were driven by a single data split.

### 2.5. Ethical and Research Approvals

This study complied with the principles of the Declaration of Helsinki and was approved by the Institutional Review Board (IRB) of Shuguang Hospital Affiliated to Shanghai University of Traditional Chinese Medicine (Approval No. 2022‐1233‐170‐01). All potential participants received an electronic study description outlining objectives, procedures, risks, and benefits and were informed that participation was voluntary and withdrawal could occur at any time without consequences. Questionnaires were completed only after obtaining online consent. To ensure confidentiality, no identifiable information was collected; all data was anonymized and used solely for academic purposes. All research team members signed confidentiality agreements to safeguard data security.

## 3. Results

### 3.1. Institutional Characteristics and MRPA Availability

A total of 123 public hospitals in Shanghai and Zhejiang Province participated in the survey, comprising 44 Tertiary Grade A hospitals (35.8%), 65 Tertiary Grade B hospitals (52.8%), and 14 Secondary Grade A hospitals (11.4%).

Departments providing MRPA in participating hospitals included Interventional Catheterization Laboratories (*n* = 50), Operating Rooms/Surgical Services (*n* = 37), Radiology Departments (*n* = 33), and Departments of Nuclear Medicine (*n* = 30). The most commonly reported MRPA items used in respondents’ departments were Lead Aprons (*n* = 99, 80.5%), Lead Thyroid Collars (*n* = 89, 72.4%), and Lead Caps (*n* = 84, 68.3%). Other items included Lead Protective Skirts (*n* = 62, 50.4%), Leaded Eyewear (*n* = 49, 39.8%), Lead Vests (*n* = 49, 39.8%), Lead Gloves (*n* = 27, 22.0%), and Lead Trousers/Pants (*n* = 22, 17.9%).

Regarding management oversight, among institutions with a designated responsible person or role for MRPA cleaning and disinfection management (*n* = 109), day‐to‐day oversight was most commonly assigned to Designated Staff Nurses (*n* = 51, 46.8%), followed by Nurse Managers/Head Nurses or Charge Nurses/Nursing Team Leaders (*n* = 38, 34.9%). Overall, nursing personnel accounted for 89 of 109 valid management‐role responses (81.7%), corresponding to 72.4% of the full sample. For the physical implementation of cleaning and disinfection, nurses were the most frequently reported executors among valid responses (*n* = 61/109, 56.0%), followed by Housekeeping/Environmental Services Staff (*n* = 33/109, 30.3%) and Physicians (*n* = 10/109, 9.2%). Detailed distributions of hospital classification, departments providing MRPA, and management roles are provided in Table 1.

**TABLE 1:** Characteristics of participating institutions and MRPA management roles (*N* = 123).

Characteristic	Category	Number (*n*)	Percentage (%)
Hospital Classification (Q1)	Tertiary Grade A	44	35.8
	Tertiary Grade B	65	52.8
	Secondary Grade A	14	11.4

Departments Providing MRPA in Participating Hospitals (Q4, multiple responses)	Interventional Catheterization Laboratory (Cath Lab)	50	40.7
	Operating Room/Surgical Services	37	30.1
	Radiology Department	33	26.8
	Department of Nuclear Medicine	30	24.4

Day‐to‐day oversight manager among institutions with designated responsible person or role (Q13, valid *n* = 109)	Department Head/Chief of Service	14	12.8
	Nurse Manager/Head Nurse	35	32.1
	Charge Nurse/Nursing Team Leader	3	2.8
	Designated Staff Nurse	51	46.8
	Other	6	5.5

Primary executor of physical cleaning/disinfection among valid responses (Q14, valid *n* = 109)	Physicians	10	9.2
	Nurses	61	56
	Housekeeping/Environmental Services Staff	33	30.3
	Other	5	4.6

### 3.2. Status and Deficiencies in Standard Practices

A total of 19.5% of institutions (24/123) reported having no unified management system for MRPA cleaning and disinfection, and 11.4% (14/123) had no designated responsible person or role. Reported deficiencies and high‐risk practices included the use of alcohol‐based disinfectants (*n* = 78, 63.4%) and chlorine‐based disinfectants (*n* = 81, 65.9%), manual cleaning and disinfection as an available cleaning/disinfection approach (*n* = 85, 69.1%), fully or partially manual traceability management (*n* = 83, 67.5%), and never having conducted quality/performance testing (*n* = 16, 13.0%).

Of the 108 institutions (87.8%) that reported providing training for cleaning/disinfection personnel, 34 (31.5%) indicated that no competency assessment or certification was conducted after training, and 14 (13.0%) reported that no periodic continuous quality improvement (CQI) training was provided. In descriptive cross‐tabulation, institutions providing training reported a higher record‐keeping rate than those without training (89.8% vs. 46.7%). Selected key deficiency indicators are summarized in Table 2.

**TABLE 2:** Key deficiencies in MRPA management practices (*N* = 123).

Deficiency domain	Deficiency indicator	*n* (%)
Management system	No unified management system for MRPA cleaning/disinfection (Q7)	24 (19.5)
	No active monitoring or management of MRPA cleaning/disinfection (Q9)	15 (12.2)
	No designated responsible person or role for MRPA cleaning/disinfection management (Q11)	14 (11.4)
	No written policies or SOPs related to MRPA management (Q30)	18 (14.6)

Processes and methods	Manual cleaning and disinfection reported as an available cleaning/disinfection approach (Q23)	85 (69.1)
	Use of alcohol‐based disinfectants (Q24)	78 (63.4)
	Use of chlorine‐based disinfectants (Q24)	81 (65.9)
	No dedicated storage area for cleaned/disinfected MRPA (Q25)	2 (1.6)
	Arbitrary storage of cleaned MRPA (Q26)	5 (4.1)

Personnel and training	No training for disinfection personnel (Q19)	15 (12.2)
	No post‐training competency assessment or certification among institutions providing disinfection‐personnel training (Q21)	34/108 (31.5)
	No periodic continuous quality improvement training among institutions providing disinfection‐personnel training (Q22)	14/108 (13.0)

Quality control and traceability	No cleaning/disinfection log or record (Q29)	19 (15.4)
	Fully or partially manual traceability management (Q33)	83 (67.5)
	Quality/performance testing conducted less than annually or never (Q34)	22 (17.9)
	Never conducted quality/performance testing (Q34)	16 (13.0)
	No formal testing report issued after quality/performance testing (Q36)	26 (21.1)

*Note:* Percentages were calculated using the full sample size (*N* = 123), except for posttraining competency assessment/certification (Q21) and periodic continuous quality improvement training (Q22), which were calculated among institutions that reported providing training for disinfection personnel (Q19 = Yes; *n* = 108).

### 3.3. Association of Institutional and Structural Frameworks With Management Practices

#### 3.3.1. Association With Hospital Grade

Hospital grade was significantly associated with the cleaning/disinfection management model (*χ* ^2^ = 22.997, *p* < 0.001). Centralized hospital‐level management was reported by 59.1% of Tertiary Grade A hospitals, 18.5% of Tertiary Grade B hospitals, and 57.1% of Secondary Grade A hospitals. Hospital grade was also associated with having a unified management system (*p* = 0.010), written policies/SOPs (*p* = 0.048), periodic CQI training (*p* = 0.012), specified sorting procedures for used or contaminated apparel (*p* = 0.035), and issuance of formal test reports after quality testing (*p* = 0.048). The proportion using quaternary ammonium compound disinfectants for rapid cleaning was lowest in Tertiary Grade B hospitals (33.8%) (*p* = 0.014).

No significant differences by hospital grade were found for having designated personnel (*p* = 0.203), providing training for disinfection personnel (*p* = 0.534), or conducting at least annual performance testing (*p* = 0.283). Personnel composition for cleaning/disinfection differed by hospital grade (*p* = 0.007); in Secondary Grade A hospitals, 28.6% reported that physicians performed these tasks. The overall distribution of departments responsible for quality testing did not differ significantly by hospital grade (*p* = 0.382).

Differences were observed in policy coverage (*p* = 0.009). As an example of policy‐content variation, outsourced transport quality‐control procedures were reported by 68.2% of Tertiary Grade A hospitals, 40.0% of Tertiary Grade B hospitals, and 57.1% of Secondary Grade A hospitals.

#### 3.3.2. Association With Cleaning and Disinfection Management Model

Centralized hospital‐level management was associated with higher proportions of having a unified management system (*p* = 0.001), designated personnel (*p* = 0.026), defined personnel role assignment (*p* = 0.009), training for disinfection personnel (*p* = 0.037), post‐training assessment/certification (*p* = 0.001), CQI training (*p* = 0.001), at least annual performance testing (*p* = 0.028), sorting processes for contaminated apparel (*p* = 0.005), and record‐keeping (*p* = 0.014). At least annual quality/performance testing was reported by 93.5% of centralized institutions, compared with 74.2% of department‐level management institutions.

In centralized hospital‐level management, cleaning/disinfection was most frequently performed by nurses (56.5%), followed by housekeeping/environmental services staff (23.9%) and physicians (15.2%). In department‐level management, nurses also represented the most common executors (48.4%), followed by housekeeping/environmental services staff (30.6%). Centralized management reported higher proportions for outsourced professional laundering, use of automated disinfection equipment (*p* = 0.001), specific rapid cleaning methods (*p* = 0.001), designated placement procedures for contaminated apparel (*p* = 0.004), and broader policy coverage (*p* = 0.001). Automated or semi‐automated cleaning/disinfection equipment was generally more frequently reported in centralized institutions than in department‐level management institutions.

Traceability differed significantly (*χ* ^2^ = 44.241, *p* = 0.001): 56.5% of centralized systems had fully electronic traceability management, compared with 3.2% in department‐level management systems. Formal testing reports were issued by 93.5% of centralized institutions and 66.1% of department‐level management institutions (*p* = 0.002). Sorting processes also differed (*p* = 0.005): centralized systems reported centralized sorting (65.2%), while department‐level management systems more often reported immediate post‐use sorting (58.1%). Training coverage was 97.8% in centralized institutions and 82.3% in department‐level management institutions (*p* = 0.037). No designated collection area or container for contaminated apparel was reported in 0% of centralized institutions and 16.1% of department‐level management institutions. Selected association statistics are presented in Table 3.

**TABLE 3:** Association of hospital classification (Q1) and cleaning/disinfection management model (Q9) on MRPA management practices.

Management practice/variable	By hospital classification (Q1)		By management model (Q9)	
	Test statistic	*p* value	Test statistic	*p* value
Unified management system for MRPA cleaning/disinfection (Q7)	Fisher’s exact	0.01	18.054	0.001
Cleaning/disinfection management model (Q9)	22.997	< 0.001	—	—
Designated responsible person or role for MRPA cleaning/disinfection management (Q11)	3.188	0.203	7.945	0.026
Primary executor of physical cleaning/disinfection (Q14)	Fisher’s exact	0.007	Fisher’s exact	0.009
Training provided for disinfection personnel (Q19)	1.347	0.534	6.949	0.037
Training content for disinfection personnel (Q20)	2.773	0.837	5.538	0.477
Post‐training competency assessment/certification for disinfection personnel (Q21)	7.042	0.134	Fisher’s exact	0.001
Periodic continuous quality improvement training for disinfection personnel (Q22)	Fisher’s exact	0.012	Fisher’s exact	0.001
MRPA cleaning/disinfection equipment (Q23)	67.818	0.001	Fisher’s exact	0.001
Rapid cleaning/disinfection methods for MRPA (Q24)	19.101	0.014	52.191	0.001
Sorting process for used MRPA (Q27)	Fisher’s exact	0.035	Fisher’s exact	0.005
Placement location of contaminated MRPA (Q28)	Fisher’s exact	0.012	Fisher’s exact	0.004
Cleaning/disinfection log or record (Q29)	2.333	0.312	8.022	0.014
Written MRPA‐related policies/SOPs (Q30)	5.963	0.048	16.76	0.001
Content coverage of MRPA‐related policies/SOPs (Q31)	29.452	0.009	69.228	0.001
Full‐process information tracking system for MRPA used in critical areas (Q32)	5.897	0.052	35.656	0.001
Form of traceability management for MRPA cleaning/disinfection (Q33)	21.957	0.001	44.241	0.001
At least annual quality/performance testing (Q34, recoded)	2.591	0.283	6.739	0.028
Department responsible for quality testing (Q35)	12.824	0.382	40.191	0.001
Issuance of formal test report after quality testing (Q36)	5.684	0.048	12.473	0.002

*Note:* Test statistics are Pearson *χ* ^2^ values unless Fisher’s exact test is indicated. For Q34, the variable was recoded as at least annual testing versus less than annual or never tested. *p* values are two‐sided.

### 3.4. Association of Personnel and Specific Policies With Implementation

#### 3.4.1. Preservice Training for Users

Of the 123 institutions, 109 reported providing preservice training for professional healthcare users. Among these, 68.8% used alcohol‐based disinfectants for rapid cleaning/disinfection, compared with 21.4% of the 14 institutions without such training (*χ* ^2^ = 10.884, *p* = 0.028).

Among institutions providing training for disinfection personnel (*n* = 108, valid analysis *n* = 97), 100% reported including “procedures and precautions for cleaning and disinfection of MRPA” as part of the training content. The proportion using dedicated storage cabinets for clean apparel was 71.6% among institutions providing preservice training and 14.3% among those without such training (*p* = 0.163). Detailed data are presented in Supporting Table 1.

#### 3.4.2. Unified Management System and Association With Practice Implementation

The presence of a unified management system was significantly associated with managerial responsibility assignments (*p* < 0.001) and personnel composition for task execution (*p* < 0.001). In institutions with such a system, dedicated nurses were more often managers (47.5% vs. 16.7%) and nurses more frequently performed cleaning/disinfection tasks (56.6% vs. 20.8%). In institutions without a unified system, the proportions without a clearly designated responsible person for oversight or physical executor were higher. Role allocation was observed to differ significantly across management and execution categories (*p* < 0.001): when department heads/chiefs of service were nominal managers, tasks were most often performed by physicians; when nursing personnel were managers, tasks were most often performed by nurses, although housekeeping/environmental services staff also accounted for a notable proportion.

The professional role allocation was also associated with quality control practices. Institutions with different personnel‐role configurations showed different distributions of quality‐testing departments, including Infection Prevention and Control or Radiology/Medical Physics departments (*p* = 0.040), and differed in whether microbial bioburden testing was included in quality‐control metrics (*p* = 0.001). Unified management was additionally associated with several specific implementation practices, including alcohol‐based rapid cleaning/disinfection, availability of ozone disinfection cabinets, and at least annual quality/performance testing. Detailed data are presented in Supporting Table 2.

#### 3.4.3. Designated Responsible Personnel and Association With Practice Implementation

Institutions with a designated responsible person or role for MRPA management reported higher training coverage for disinfection personnel (95.4% vs. 28.6%) and higher rates of CQI training (85.3% vs. 7.1%) (Fisher’s exact test, *p* < 0.001). Record‐keeping rates were 91.7% in institutions with a designated responsible person or role and 28.6% in those without (*p* < 0.001).

In quality control, 88.1% of institutions with a designated responsible person or role reported conducting at least annual quality/performance testing, compared with 35.7% of institutions without such a role (*p* < 0.001). Detailed results are shown in Supporting Table 3.

### 3.5. Core Role and Association of Nursing Personnel With MRPA Management

#### 3.5.1. Association of Institutional Policies With Nursing Roles

Cross‐tabulation showed a significant association between the presence of a unified management system and personnel role allocation (*p* < 0.001). In institutions with a unified system, dedicated nurses were more often managers (47.5% vs. 16.7%), and nurses were more frequently the primary executors of cleaning/disinfection tasks (56.6% vs. 20.8%). In institutions without a unified system, task allocation was more dispersed, with a higher proportion lacking a clearly designated responsible person or physical executor.

Role allocation differed significantly across management and execution categories (*p* < 0.001): when department heads/chiefs of service were designated managers, execution was most often performed by physicians; when nursing personnel were managers, execution was more often carried out by nurses, although housekeeping/environmental services staff also accounted for a notable proportion. Professional role allocation was associated with quality control pathways and selected indicators. Institutions with different personnel‐role configurations showed different distributions of quality‐testing departments, including Infection Prevention and Control or Radiology/Medical Physics departments (*p* = 0.040), and differed in whether microbial bioburden was included in quality‐control metrics (*p* = 0.001). Detailed data are presented in Supporting Table 2.

#### 3.5.2. Association of Nurses as Executors With Key Process Stages

Analyses by executor category showed no significant association with equipment selection (*p* = 0.795) or rapid cleaning/disinfection method (*p* = 0.657). However, the executor category was significantly associated with the department responsible for quality testing (*p* = 0.040) and the selection of testing indicators (*p* = 0.001). In this analysis, nurse execution was examined as one executor category rather than as an isolated binary exposure. Detailed data are presented in Supporting Table 4.

#### 3.5.3. Associations of Nursing‐Related Management Roles With Selected MRPA Practices

When nurses served as managers or when MRPA management systems were under the Nursing Department, higher performance was reported across selected dimensions. Record‐keeping rates differed significantly across manager categories (*p* < 0.001), with the highest rates observed when the manager was a nurse manager/head nurse or charge nurse/nursing team leader (100.0%), followed by designated staff nurses (88.2%). When no designated responsible person was identified, the record‐keeping rate was substantially lower (28.6%). The proportion of institutions conducting post‐training competency assessment/certification also differed significantly across manager categories (*p* < 0.001), with markedly lower implementation when no designated responsible person was identified.

In quality control, institutions with an identifiable managerial role, including nursing‐related managers, had higher rates of at least annual quality/performance testing (*p* = 0.002). Institutions under the Nursing Department more often had nurses performing cleaning tasks (71.4% vs. 48.6%, *p* = 0.015) and more frequently assigned the Nursing Department as the quality testing department (36.7% vs. 8.1%, *p* < 0.001).

These institutions reported higher rates of training for disinfection personnel (*p* = 0.027) and CQI training (*p* = 0.029). No significant differences were observed for formal test‐report issuance or for the inclusion of attenuation‐property or physical‐integrity testing indicators in the Nursing Department affiliation analysis. Detailed data are shown in Supporting Table 5.

### 3.6. Model Performance and Robustness Checks

Predictive Performance (Supporting Table 6): We evaluated three distinct machine learning algorithms on an independent test set to compare predictive performance. Model performance was assessed using AUC‐receiver operating characteristic (ROC), accuracy, sensitivity/recall, specificity, positive predictive value (PPV), negative predictive value (NPV), balanced accuracy, Cohen’s kappa, and Brier score. As detailed in Supporting Table 6, the random forest model demonstrated the most balanced performance, achieving an accuracy of 75.0% and a sensitivity of 75.9%. Notably, the PPV was 91.7%, indicating a high proportion of true CNP cases among institutions classified as high risk in this test set. The consistency across algorithms (e.g., LASSO also achieving a PPV of 91.3%) supports the robustness of the observed predictive pattern.

Proxy Analysis of Inspection Frequency (Supporting Table 7): To examine whether inspection frequency was related to the elevated CNP prevalence observed in Tertiary Grade A hospitals, we analyzed the relationship between “Quality Inspection Frequency” (a proxy for management intensity) and disinfection practices. Supporting Table 7 reveals no significant association between inspection frequency and improper disinfection rates across the examined hospital strata (Tertiary A: *p* = 0.882; Tertiary B: *p* = 0.280; Overall: *p* = 0.395). In Tertiary A hospitals, the noncompliance rate remained consistently high (> 90%) regardless of whether inspections were monthly or annual. This proxy analysis did not support inspection frequency as an explanation for the observed pattern, although unmeasured workload indicators could not be fully excluded. The predictive capability of the models is illustrated by the ROC curves shown in Figure 1, where the random forest model showed the most balanced overall performance.

**FIGURE 1:**
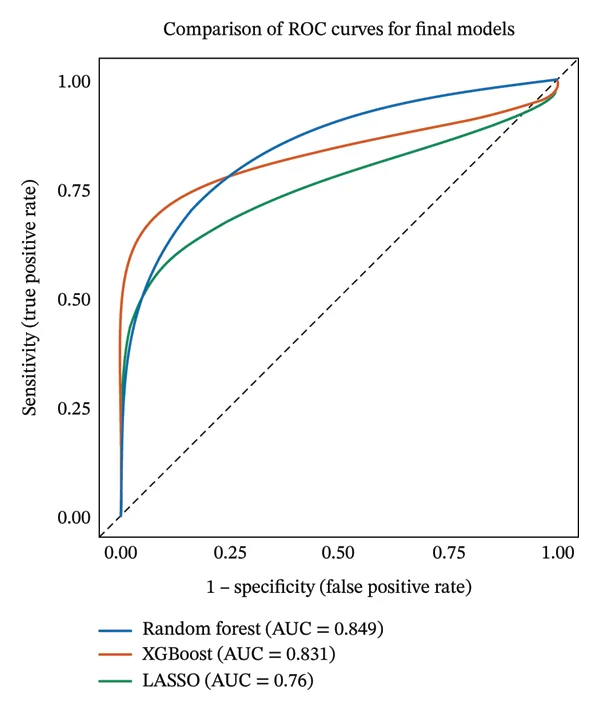
Comparison of receiver operating characteristic (ROC) curves. Note: the plot displays the performance of the random forest, XGBoost, and LASSO models on the independent test set.

#### 3.6.1. Global Feature Importance for Predicting CNP

A Random Forest classification model was developed and validated to examine organizational factors associated with CNP. Predictor importance was ranked using permutation importance (Figure 2A). The highest‐ranked predictor was hospital classification: Tertiary Grade A, followed by the presence of a traceability system for MRPA in critical areas. The remaining top five predictors were MRPA cleaning/disinfection performed by housekeeping/environmental services staff, the presence of designated personnel for MRPA cleaning/disinfection management, and MRPA cleaning/disinfection performed by a third‐party laundry service.

**FIGURE 2:**
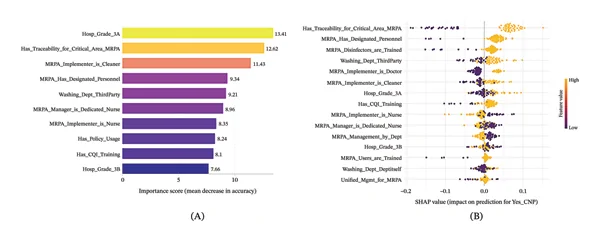
Global feature importance for predicting CNP. Note: global feature importance analysis: (A) permutation‐based importance ranking: features are ranked by their average impact on the model’s accuracy. (B) SHAP summary plot: this displays the distribution of each feature’s impact on the model’s prediction for all samples. The color of the points represents the feature’s original value.

SHAP summary plots (Figure 2B) were used to assess feature contributions to model predictions. Positive SHAP values indicate an association with a higher predicted probability of CNP, whereas negative values indicate an association with a lower predicted probability. Ranking by mean absolute SHAP value identified the presence of a traceability system for MRPA in critical areas, the presence of designated personnel, and disinfection personnel having received training as the features with the highest contributions to the model output.

For the presence of a traceability system for MRPA in critical areas, higher feature values (“Yes”) were predominantly located on the positive SHAP side, indicating a higher predicted probability of CNP in the model. Similar patterns were observed for the presence of a designated responsible person or role, training for disinfection personnel, and MRPA cleaning/disinfection performed by a third‐party laundry service, where higher feature values were mostly linked to positive SHAP values. In contrast, nurse‐related variables, including MRPA cleaning/disinfection performed by nurses and MRPA cleaning/disinfection manager being a dedicated nurse, showed more heterogeneous or near‐zero SHAP distributions.

For Hospital Classification: Tertiary Grade A, higher feature values were also largely associated with positive SHAP values. MRPA cleaning/disinfection performed by housekeeping/environmental services staff also showed a generally positive SHAP pattern. These findings suggest that several system‐enabling variables were associated with higher predicted CNP risk in this dataset, supporting the interpretation that guideline‐discordant practices may have become embedded within more structured management environments rather than reflecting simple absence of governance.

#### 3.6.2. Interaction Analysis: Governance Structure and Preservice Training

SHAP analysis was used to examine the interaction between unified management of MRPA cleaning/disinfection and MRPA users who have received preservice training. Figure 3A shows the mean SHAP contribution values of the “preservice training for MRPA users” feature to the model’s prediction of CNP, stratified by governance model.

**FIGURE 3:**
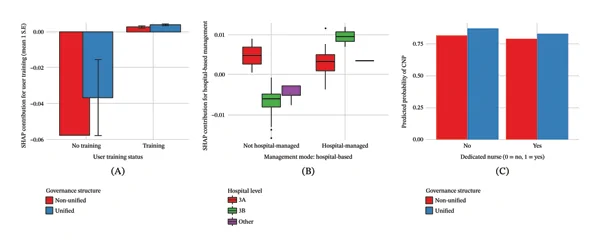
Interaction effects of key organizational factors on compliance risk. Note: this figure illustrates complex interactions between key organizational factors, analyzed via SHAP values and predicted probabilities. (A) Governance and training interaction: average contribution of “user training” to risk prediction (mean SHAP value). (B) Management and hospital tier interaction: contribution distribution of “hospital‐wide management” to risk prediction (SHAP value distribution). (C) Dedicated nurse and governance structure interaction: model’s predicted probability of noncompliance for each combination.

In institutions with nonunified management, the absence of preservice training was associated with negative mean SHAP values (−0.055). A similar but less pronounced negative pattern was observed in unified management settings. In contrast, in both unified and nonunified management settings, the presence of preservice training was associated with positive SHAP values.

#### 3.6.3. Interaction Analysis: Hospital Classification and the Association With Management Model

The potential moderating effect of hospital classification on the relationship between hospital‐wide MRPA cleaning/disinfection management and predicted CNP was examined. Figure 3B presents the SHAP value distributions for this management model, stratified by hospital classification.

In Tertiary Grade B hospitals, nonhospital‐wide management was associated with SHAP values mainly in the negative range, whereas hospital‐wide management showed SHAP values with a median above zero and predominantly in the positive range. In Tertiary Grade A hospitals, SHAP values for both hospital‐wide management and nonhospital‐wide management were mainly distributed in the positive range.

#### 3.6.4. Interaction Analysis: Dedicated Nurse and Governance Structure

The interaction between the MRPA cleaning/disinfection manager, who is a dedicated nurse, and the unified management of MRPA cleaning/disinfection was examined by calculating predicted probabilities of CNP for four scenarios, holding all other variables at their median or mode values (Figure 3C).

Across all four scenarios, the predicted probability of CNP ranged from 78% to 85%. Compared with the baseline scenario (no dedicated nurse and nonunified management), the presence of a dedicated nurse alone showed minimal difference in predicted probability. Scenarios with unified management showed slightly higher predicted probabilities than the nonunified baseline, regardless of whether a dedicated nurse was present. These findings suggest that the dedicated nurse role alone did not substantially offset the predicted risk pattern associated with the broader governance structure.

#### 3.6.5. Case‐Level SHAP Attribution: Representative Examples

In addition to global feature importance, SHAP analysis was used to explain the prediction logic for individual cases. Case‐level SHAP attribution plots (Figure 4) illustrate the leading local contributions for two representative hospitals, including one best‐performing compliant case and one worst‐performing noncompliant case.

**FIGURE 4:**
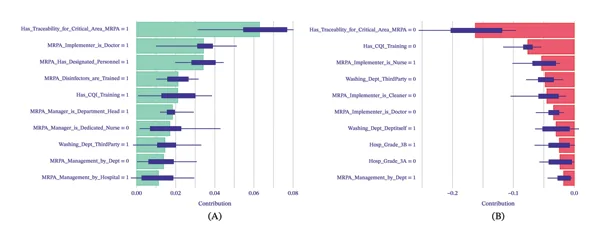
Case‐level SHAP attribution plots for local interpretability of two representative cases. Note: case‐level SHAP attribution plots illustrate the model’s local prediction logic for two representative cases: (A) a best‐performing compliant case and (B) a worst‐performing noncompliant case. Bars represent local feature contributions for the displayed cases.

Figure 4A shows a representative hospital correctly predicted as compliant. The local contribution profile was mainly characterized by structured management and implementation features. The largest displayed contribution was from the presence of a traceability system for MRPA in critical areas. Additional leading contributors included MRPA cleaning/disinfection performed by physicians, presence of designated personnel for MRPA management, training for disinfection personnel, and CQI training.

Figure 4B presents a representative hospital correctly predicted as noncompliant. The local contribution profile was dominated by the absence of key system‐enabling features. The largest displayed contribution was associated with the absence of a traceability system for MRPA in critical areas. Other leading contributors included the absence of CQI training, MRPA cleaning/disinfection performed by nurses, the absence of third‐party laundering, and the absence of housekeeping/environmental‐services execution.

To provide a more explicit numerical example of local attribution, an additional SHAP waterfall plot for another representative institution is provided in Supporting Figure 1. In this example, the model baseline value was *E*[*f*(*x*)] = 0.803, and the final predicted probability was *f*(*x*) = 0.862. The largest positive contribution was from the presence of critical‐area traceability (+0.094), followed by housekeeping/environmental‐services staff as executors (+0.020), in‐house laundering (+0.0148), and the absence of a dedicated nurse manager (+0.0145). Negative contributions included absence of third‐party laundering (−0.0468), non–Tertiary Grade A status (−0.0258), and absence of department‐level laundering (−0.0138). Together, these representative examples suggest that local predictions may reflect combinations of traceability infrastructure, executor allocation, laundering pathway, and management‐role variables rather than a single isolated factor.

To further analyze the random forest model beyond single‐dimension ranking, a multidimensional feature importance plot (Figure 5) was used. This plot integrates several complementary metrics to provide a richer view of variable importance. In Figure 5A, mean_min_depth represents a variable’s average splitting depth across decision trees, with lower values indicating earlier use in tree construction, whereas times_a_root indicates how frequently a variable was selected as the primary splitting node. In Figure 5B, accuracy_decrease and gini_decrease provide complementary measures of predictive contribution and node impurity reduction.

**FIGURE 5:**
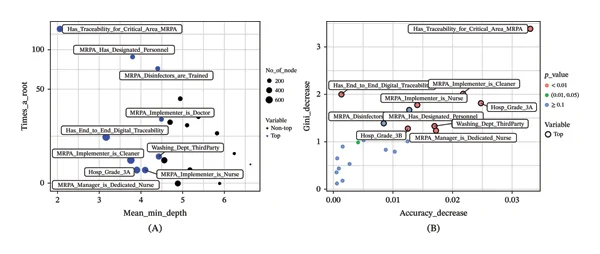
Multidimensional random forest importance plot visualizing variable importance and split structure. Note: this multidimensional importance plot summarizes random forest variable importance using multiple metrics. (A) Integrates mean minimal depth and times as a root node; lower mean minimal depth and higher root‐node frequency indicate earlier and more frequent use in tree construction. (B) Integrates accuracy decrease and Gini decrease; variables positioned further toward the upper‐right indicate stronger combined importance. Point color denotes the *p* value category, and outlined points indicate top‐ranked variables.

Across these multidimensional importance metrics, the presence of a traceability system for MRPA in critical areas emerged as the most influential variable. It showed both high root‐node frequency and strong importance in terms of accuracy decrease and Gini decrease. The presence of designated personnel and training for disinfection personnel also showed high root‐node frequency, suggesting that personnel assignment and training‐related variables were repeatedly used in early model splits. Executor‐related variables, including cleaning/disinfection performed by housekeeping/environmental‐services staff and nurses, as well as hospital classification and third‐party laundering, contributed across different importance dimensions.

Overall, the multidimensional importance pattern indicated that multiple feature domains—including traceability infrastructure, personnel assignment, executor composition, training, laundering pathway, and institutional context—contributed to CNP prediction, rather than a single management‐role variable.

## 4. Discussion

MRPA is essential for ensuring occupational safety in ionizing radiation environments, yet our findings suggest a substantial gap between established standards and routine practice. In our study, nearly two‐thirds of institutions reported using alcohol‐based or chlorine‐based disinfectants for rapid cleaning/disinfection (63.4% and 65.9%, respectively), and 67.5% relied on fully or partially manual traceability management, limiting quality assurance and lifecycle oversight. Governance gaps were also evident: 19.5% of institutions lacked a unified management system, and 11.4% had no designated responsible person or role. The coexistence of guideline‐discordant disinfection, incomplete digital traceability, and gaps in formal responsibility indicates that the observed guideline–practice gap extended across multiple stages of the MRPA management pathway rather than being confined to a single technical practice.

Comparable deficiencies have been reported across different dimensions of MRPA management internationally, although the populations and outcomes assessed are not directly comparable. In a U.S. survey of healthcare workers, predominantly registered nurses, 52.0% were unsure how spot cleaning was completed, and 28.3% reported that their hospital or facility had no policy on spot cleaning [11]. By contrast, a multicenter study in Togo assessed lead aprons as the unit of analysis using physical, radiographic, and dosimetric evaluation. In that study, 62.79% of the aprons showed radiographic structural defects, while 46.51% were stored randomly and 16.28% were folded [3]. These studies therefore reflect different levels of the MRPA management pathway—staff‐reported cleaning and policy implementation in the U.S. study and equipment condition and storage in the Togo study—rather than directly comparable prevalence estimates. MRPA has also been identified as an overlooked contamination source in operating rooms [7], suggesting that management of protective apparel may receive less attention than its protective function. Although previous studies have often emphasized unclear responsibilities or limited staff awareness, these explanations capture only part of the implementation problem. Implementation research increasingly recognizes guideline uptake as embedded within a broader organizational context, including leadership, communication, resources, monitoring, and feedback processes [28].

The machine‐learning analyses further supported this systems‐level interpretation. Random forest modeling with SHAP analysis indicated that CNP prediction reflected contributions from multiple organizational and workflow features rather than a single professional role or isolated management label. Variables related to institutional tier, critical‐area traceability, designated responsibility, disinfection‐personnel training, executor composition, and laundering pathway repeatedly contributed to prediction. This pattern suggests that guideline‐discordant disinfection behavior may be better explained by infrastructure, workflow configuration, and institutional context than by professional role alone. It is also consistent with broader literature emphasizing governance, digitization, and appropriate technology as hallmarks of high‐reliability healthcare organizations [29].

These findings suggest a deeper challenge: institutions may be balancing two competing risk management logics—long‐term asset preservation, aligned with international standards, versus short‐term infection control, shaped by local constraints and risk perception. A parallel can be drawn from antibiotic stewardship, where guideline‐concordant prescribing is often deprioritized under pressure to manage immediate clinical risks [30]. Similarly, MRPA noncompliance may reflect pragmatic decisions to address immediate contamination concerns, even at the expense of long‐term equipment integrity and occupational safety. Infection‐related risks are immediate and emotionally salient to clinical staff [31, 32], whereas equipment degradation and diminished radiation protection are long‐term and less perceptible. This imbalance in risk salience may contribute to greater attention toward short‐term threats.

However, this trade‐off may also be shaped by the local knowledge environment, although exposure to specific expert consensus documents was not directly measured in this study. An anomalous training‐related signal further supports this possibility: institutions providing training showed a higher proportion of nonrecommended disinfectant use than those without. While this descriptive difference is statistically unstable due to sample imbalance, it prompted further analysis. Among institutions with valid training‐content responses, all reported covering procedures and precautions for MRPA cleaning and disinfection, suggesting that training content and implementation context may be important for understanding this pattern. Because the questionnaire did not capture the specific sources of training content, the link between institutional training and local expert consensus should be interpreted as inferential. Nevertheless, when training content is aligned with guidance that differs from manufacturer‐aligned material‐care recommendations, training may reinforce guideline‐discordant practices rather than correct them.

One possible explanation concerns the content and implementation context of training. Most institutions provided training for cleaning/disinfection personnel, posttraining competency assessment, and periodic CQI training were not universal, suggesting that training quality and feedback mechanisms remained variable. At the same time, domestic specialty guidance and manufacturer‐aligned material‐care recommendations provide different signals for frontline implementation. China’s MRPA governance illustrates a “hierarchy paradox”: the Chinese National Health Industry Standard WS 310.2‐2016 [14], which emphasizes adherence to manufacturers’ instructions, coexists with widely adopted consensus statements [17, 18] that deviate significantly. For example, a “best evidence summary” designates alcohol‐ and chlorine‐based disinfection as A‐level recommendations. A more influential consensus also lists such products as routine disinfection options, arguing that manufacturer‐recommended products are often inaccessible and framing lead apparel as a consumable item. These documents promote an approach framed as economical and efficient, reflecting broader policy pressures such as China’s volume‐based procurement (VBP) strategy, which has drastically reduced the cost of high‐value consumables since 2018. In this context, following consensus‐supported disinfection options may appear operationally rational: medical alcohol wipes cost RMB 13, and chlorine wipes cost RMB 25–35, while adhering to the international “gold standard” is prohibitively expensive manufacturer‐endorsed wipes may exceed RMB 400. Therefore, the observed divergence from manufacturer‐aligned recommendations may reflect context‐dependent adaptation to local operational and economic constraints, rather than individual‐level negligence alone.

Nonetheless, the consensus diverges from current international evidence. Its conclusion of “limited damage” appears to emphasize evidence suggesting limited material harm, while simulation studies have also reported degradation after repeated alcohol‐ or chlorine‐containing wiping [16]. It also rates lead caps as offering only mild protection (Grade B, 5C), which differs from recent studies demonstrating their efficacy [33–35]. In this context, the observed training‐related pattern raises the hypothesis that, where institutional training adopts similar recommendations, formal training could reinforce guideline‐discordant practices and confer a degree of institutional legitimacy on them. This proposed mechanism was not directly assessed in the present study and should be examined in future research through direct assessment of training content, information sources, and subsequent practice adoption. The observed 68.3% lead cap use rate in respondent departments may reflect how such evidence is translated into local practice, although item availability or use alone does not ensure standardized cleaning, storage, or performance monitoring. Even well‐designed interventions are unlikely to succeed without supporting infrastructure [35]. These findings are consistent with High‐Reliability Organization (HRO) theory, which emphasizes robust governance, technology‐supported error prevention, and continuous feedback loops as essential for sustained safety [36].

A second notable pattern identified by the model is that Tertiary Grade A hospital status was the highest‐ranked predictor in permutation importance analysis (Figure 2A) and showed a generally positive SHAP pattern in Figure 2B, challenging assumptions that higher‐tier hospitals inherently demonstrate superior management due to greater resources [37]. Chi‐square analyses confirmed that these institutions excel in establishing centralized governance and policy frameworks. However, these strengths did not translate into significantly higher rates of designated responsible personnel or at least annual performance testing, suggesting that stronger formal governance did not necessarily coincide with more complete operational implementation.

Furthermore, our sensitivity analysis (Supporting Table 7) did not support inspection frequency as the primary explanation for the observed positive predictive contribution of Tertiary Grade A status. The lack of association between inspection frequency and improper disinfection rates in Tertiary Grade A hospitals, together with the nonsignificant overall association, suggests that inspection frequency alone is unlikely to explain this pattern. However, hospital grade may also capture a broader cluster of institutional characteristics that were not directly measured in this study. Tertiary Grade A hospitals typically operate within more complex organizational environments and may differ in patient and procedure volume, clinical workload, case mix, departmental breadth, staffing patterns, equipment turnover, and cross‐departmental coordination. Inspection frequency does not adequately represent these dimensions. Consequently, unmeasured service volume, workload, institutional complexity, and other correlated organizational characteristics may have contributed to the predictive importance attributed to Tertiary Grade A status. The present analysis cannot disentangle these correlated features from hospital grade itself. The positive SHAP pattern for Tertiary Grade A status should therefore be interpreted as a predictive signal associated with the broader institutional context rather than as evidence that hospital tier independently increases guideline‐discordant practice. One additional hypothesis, discussed below, is that professional guidance and institutional information pathways may also contribute to this pattern.

Two distinct failure patterns emerged (Figure 3B). In Tertiary Grade B hospitals, hospital‐wide management was associated with a more positive SHAP contribution to predicted CNP risk, whereas nonhospital‐wide management showed mainly negative SHAP values. One possible interpretation of this pattern is a form of policy–practice decoupling, in which centralized management structures are formally established but are not fully translated into routine operational practice. This possibility is conceptually consistent with “symbolic adoption”; however, the present study did not directly assess the motivations for adopting centralized management, external mandate pressures, resource sufficiency, or implementation capacity, and therefore cannot determine whether symbolic adoption actually occurred. More broadly, research on fragmented healthcare systems has emphasized that formal organizational structures alone may be insufficient when cross‐departmental coordination and learning remain limited, highlighting the importance of breaking organizational silos and strengthening collaboration across boundaries [38]. Similar phenomena have been reported in the management of other high‐risk equipment, such as flexible endoscopes: despite strict guidelines, complex workflows have led even leading medical centers to frequently report noncompliant practices [21].

In Tertiary Grade A hospitals, SHAP values were mainly positive regardless of whether hospital‐wide management was present. One hypothesis raised by this pattern is a possible authority–influence misalignment, whereby formal governance structures may not necessarily align with the sources of professional influence shaping technical routines. However, professional influence and the adoption or dissemination of routines perceived as authoritative were not directly measured in this study; this interpretation is therefore hypothesis‐generating and requires further investigation. Previous evidence indicates that local opinion leaders can influence professional practice and guideline uptake [39], providing a plausible basis for future studies to examine whether similar influence pathways operate in MRPA management. A key paradox identified by the SHAP analysis concerns the role of training: the presence of training for disinfection personnel was generally associated with a positive SHAP contribution to predicted CNP risk, rather than acting as a uniformly protective factor. This finding challenges binary notions of training as inherently beneficial and suggests that training may function as an organizational amplifier, with its association with outcomes shaped by the institutional environment in which it is embedded.

In low‐capacity settings, where quality‐control mechanisms are limited and training content aligns with guideline‐discordant recommendations, training may act as a risk accelerator. It may standardize flawed procedures through formal dissemination channels, allowing error‐prone workflows to become embedded in routine practice [40]. In contrast, in institutions with strong internal error‐correction capacity, the positive aspects of training may be filtered and validated. In the minority of high‐performing institutions—those with strong internal governance structures (e.g., centralized management and traceability systems)—an “evidence filter” may operate. Even when training content is flawed, positive elements such as emphasis on the importance of management and process standardization may be retained, while recommendations that diverge from manufacturer‐aligned material‐care guidance are more likely to be identified, filtered, and corrected by authoritative internal quality or infection control departments. This duality is reinforced by SHAP’s individual attribution analysis. In the representative noncompliant case shown in Figure 4B, the local attribution profile highlighted the absence of key system‐enabling features, including the absence of critical‐area traceability and absence of CQI training, together with executor and laundering‐pathway variables.

Nursing leadership literature describes nurses and nurse leaders as an important nexus between organizational structures and frontline safety practices [41]. In our study, nursing personnel accounted for the majority of valid management‐role responses (81.7%), and nurses were the most frequently reported physical executors of cleaning/disinfection among valid responses (56.0%). These findings indicate that nurses occupied a central position in the MRPA management pathway, both as coordinators and as frontline implementers. However, nursing involvement should not be equated with role clarity or system reliability. International evidence suggests that MRPA‐related responsibilities are often ambiguously defined: some staff are unaware of whether cleaning policies exist, nurses’ perceived management duties vary widely, and cleaning responsibilities may shift between surgeons, nurses, and other personnel [7, 11, 42]. This ambiguity suggests that the Nursing Nexus may function either as a coordination hub or as a site where poorly specified responsibilities are transferred and normalized. Therefore, the central role of nurses in MRPA management should be understood as a structural position requiring clear authority, standardized workflows, and institutional support, rather than as evidence that nursing participation alone ensures compliance.

This raises a central question: if nurses are already heavily involved, why does management failure persist? The model findings suggest that nursing participation alone does not account for the observed CNP pattern. Although nurses occupied a central role in both management and implementation, nurse‐related variables showed comparatively limited or heterogeneous contributions to model prediction, whereas organizational and workflow characteristics—including traceability, designated responsibility, training, hospital tier, executor composition, and laundering pathways—contributed more substantially. This interpretation is consistent with Kanter’s Structural Empowerment Theory [43]. These findings do not diminish the significance of nurses but emphasize that empowerment, not just participation, is critical for complex organizational change [44]. Kanter’s framework identifies access to Opportunity, Information, Support, and Resources as core structural conditions of workplace empowerment [43]. These constructs were not directly measured in the present study; rather, we use the framework as an interpretive lens for the organizational characteristics captured by our survey. Within this theoretical mapping, clear managerial authority and designated responsible roles may correspond conceptually to Support; traceability systems to Information; and standardized laundering pathways, equipment, and quality‐control capacity to Resources. Training‐related variables may overlap conceptually with Opportunity for learning and development, but Opportunity as a theoretical construct was not directly assessed. These correspondences should therefore be understood as conceptual mappings of the observed organizational variables rather than direct measurements of structural empowerment. Prior nursing research has linked structural empowerment with greater work engagement and organizational commitment among nurses [45].

This distinction between role presence and the conditions surrounding that role is also reflected in Figure 3C. The presence of a dedicated nurse manager alone did not clearly differentiate predicted CNP across the modeled scenarios. Because managerial authority, decision‐making latitude, access to information and resources, and organizational support were not directly assessed, this pattern is better understood as reflecting the organizational context in which the role operates than as evidence regarding the effectiveness of nurse managers themselves. Future research should examine how these contextual conditions modify the contribution of dedicated nursing‐management roles to MRPA implementation.

The distribution of MRPA responsibilities across professional groups adds another layer to this organizational context. Role allocation differed significantly across managerial and executor categories. When department heads/chiefs of service were designated managers, execution was most often performed by physicians; when nursing personnel were managers, execution was more often carried out by nurses, although housekeeping/environmental‐services staff also accounted for a notable proportion. These patterns indicate substantial heterogeneity in how MRPA management and cleaning responsibilities are organized across institutions. They do not, however, establish that task shifting or de‐skilling occurred, because the study did not assess whether responsibilities had been transferred between professional groups, the task‐specific competency of individual executors, or safety outcomes associated with different staffing arrangements. MRPA‐specific studies have likewise reported unclear cleaning responsibilities and inconsistent sanitation practices among healthcare workers [11, 42]. The more directly supported concern is therefore not which professional group performs the task, but whether responsibility is clearly assigned and accompanied by appropriate competency, standardized procedures, and organizational oversight.

These role‐allocation findings fit within the broader organizational pattern identified by the model. CNP predictions reflected contributions from traceability infrastructure, designated personnel, disinfection‐personnel training, executor composition, hospital tier, and laundering pathway rather than a single professional role or management label. The random forest–SHAP analysis characterized the contribution of these features to model predictions rather than estimating independent effect sizes, while case‐specific SHAP profiles showed that similar predicted probabilities could arise from different combinations of organizational and workflow features. This heterogeneity suggests that future intervention research should move beyond isolated management components and evaluate coordinated strategies combining explicit accountability, traceability, standardized laundering and disinfection pathways, and competency‐based quality improvement.

More broadly, this study provides an institution‐level view of MRPA implementation that complements previous work focused primarily on equipment condition, contamination, or individual practice. By combining multicenter organizational data with interpretable machine‐learning analysis, it identifies recurrent predictive patterns across responsibility assignment, training, traceability, executor allocation, and laundering pathways. In a setting where institutions may encounter discordant sources of technical guidance, these findings define specific organizational domains for prospective validation and for testing whether coordinated changes in management structure, training content, traceability, and workflow improve guideline‐concordant MRPA practice.

## 5. Limitations

This study has several limitations that define its exploratory nature. First, concerning generalizability, we used convenience sampling from public hospitals at the secondary A level or above in Shanghai and Zhejiang Province. Because no probability‐based regional sampling frame was used, the representativeness of the participating institutions within these two regions cannot be established. The findings, therefore, should not be generalized to all hospitals in Shanghai or Zhejiang or to less developed regions of China, resource‐limited hospitals, rural healthcare settings, or healthcare systems with different organizational structures and regulatory environments. The cross‐sectional design precludes causal inference, and the random forest–SHAP analyses should be interpreted as explanations of model prediction and statistical association rather than causal mechanisms. Data were based on self‐reported questionnaires completed by a single informant per hospital, which may introduce reporting bias, including social desirability bias, and may not fully capture interdepartmental heterogeneity. Direct observation, document review, manufacturer verification, or physical inspection of MRPA was not performed. In addition, the binary definition of CNP simplified complex practice patterns, including disinfection frequency, product concentration, exposure duration, operational intent, and cumulative equipment damage. Finally, interpretations of the Tertiary Grade A predictive pattern, expert consensus, procurement constraints, and VBP, may be influenced by unmeasured contextual factors and should therefore be regarded as inferential. Future longitudinal and mixed‐methods research is needed to validate these findings and clarify the systemic barriers to guideline‐concordant MRPA management.

## 6. Conclusion

In conclusion, this multicenter study identified substantial variation in MRPA disinfection, traceability, and management practices across public hospitals in Shanghai and Zhejiang Province. Interpretable machine‐learning analyses indicated that CNP prediction reflected a combination of hospital tier, traceability, designated responsibility, training, executor composition, and laundering pathways rather than a single professional role or management feature. Nurses occupied a central position in both management and implementation, but their contribution was embedded within these broader organizational arrangements. Prospective studies should validate these patterns and test whether coordinated changes in accountability, evidence‐aligned training, traceability, and standardized workflow improve guideline‐concordant MRPA management.

## Author Contributions

Suwen Li: conceptualization, data curation, formal analysis, investigation, methodology, project administration, resources, software, supervision, validation, visualization, writing–original draft, and writing–review and editing. Xing Xin: conceptualization, investigation, methodology, software, writing–original draft, and writing–review and editing. Chenxin Ye: data curation, investigation, methodology, validation, and writing–review and editing. Yingchen Lu: investigation, data curation, methodology, validation, and writing–review and editing. Yuting Wu: investigation, data curation, methodology, Validation, and writing–review and editing. Gendi Lu: conceptualization, investigation, project administration, resources, supervision, and writing–review and editing.

## Funding

This work was funded by the University‐Bureau Level Scientific Research Project of Shuguang Hospital Affiliated to Shanghai University of Traditional Chinese Medicine (No. 2023WB05).

## Ethics Statement

This study was conducted in accordance with the ethical principles of the Declaration of Helsinki. The research protocol was approved by the Institutional Review Board (IRB) of Shuguang Hospital Affiliated to Shanghai University of Traditional Chinese Medicine (Approval No. 2022‐1233‐170‐01), with initial approval granted on December 28, 2022, and a 1‐year extension approved on December 1, 2024.

All potential participants received an electronic study description outlining the purpose, procedures, risks, and benefits. They were informed that participation was entirely voluntary and that they could withdraw at any time without adverse consequences. Questionnaires were completed only after obtaining online consent. To ensure privacy, the questionnaire was designed to avoid collecting any identifiable information, and all data were anonymized and used solely for the academic purposes of this study. All members of the research team signed confidentiality agreements to ensure data security.

## Consent

Please see the Ethics Statement.

## Conflicts of Interest

The authors declare no conflicts of interest.

## Supporting Information

Additional supporting information can be found online in the Supporting Information section.

## Supporting information


**Supporting Information 1** Supplementary Figure 1. SHAP Waterfall Plot for Local Attribution in a Representative Institution.

**Supporting Information 2** Supplementary Table 1. Multiple Response Chi‐square Analysis of Disinfection and Storage Methods by Preservice Training Status.

**Supporting Information 3** Supplementary Table 2. Association between Unified Management System, Personnel‐Role Allocation, and MRPA Implementation Practices.

**Supporting Information 4** Supplementary Table 3. Impact of “Designated Personnel” on Key MRPA Management Practices.

**Supporting Information 5** Supplementary Table 4. Association between Executor Category and Key MRPA Process Stages.

**Supporting Information 6** Supplementary Table 5. Associations of Nursing‐Related Management Roles with Selected MRPA Practices.

**Supporting Information 7** Supplementary Table 6. Performance Comparison of Machine Learning Models in the Independent Test Set.

**Supporting Information 8** Supplementary Table 7. Proxy Analysis of the Association between Quality Inspection Frequency and Improper Disinfection Rates.

**Supporting Information 9** Supplementary File 1. Complete Survey Instrument for the Management, Cleaning, and Disinfection of Medical Radiation Protective Apparel.

**Supporting Information 10** Supplementary File 2. Mapping Table of Survey Items to Machine Learning Features.

**Supporting Information 11** Reporting checklist for cross‐sectional study.

## Data Availability

The data that support the findings of this study are available from the corresponding author upon reasonable request.
