# Supplementary material for: Organizational and Nursing Management Factors Associated With Guideline‐Discordant Medical Radiation Protective Apparel Disinfection: A Multicenter Interpretable Machine Learning Study
Source: J Nurs Manag. 2026 Nov 8;2026:2036458. doi: 10.1155/jonm/2036458 (PMC13745874; doi:10.1155/jonm/2036458)
Supplement: Supplementary file 2 [file JONM-2026-2036458-s008.docx]

# A Survey Instrent for the Management, Cleaning, and Disinfection of Medical Radiation Protective Apparel: A Framework for Quality Assurance and Regulatory Compliance

## Part I: To our valued participant

Thank you for contributing to this important study. This survey is designed to assess the full spectrum of policies and practices regarding the management of medical radiation protective apparel (MRPA) within healthcare institutions. Your expert input is crucial for developing a comprehensive understanding of current standards and identifying opportunities for enhancing the safety of both patients and healthcare personnel.

Medical radiation protective apparel, such as lead aprons and thyroid collars, is unique in that it represents a critical intersection of two fundamental safety domains: radiation protection and infection prevention. While its primary function is to shield against ionizing radiation, its nature as a high-touch, reusable clinical item creates a significant risk of it becoming a vehicle for healthcare-associated infections (HAIs). Therefore, effective management requires a comprehensive approach that encompasses procurement, user training, daily handling, scientifically validated cleaning and disinfection protocols, regular integrity testing, and compliant disposal.

Your expertise will help create a clear picture of the procedures and protocols that govern the entire lifecycle of this essential equipment. The data gathered will be invaluable for informing best practices, guiding policy development, and ensuring readiness for regulatory audits from bodies such as The Joint Commission.

Please answer each question to the best of your knowledge. We appreciate you taking the time to provide detailed and accurate information. Your participation is a vital contribution to advancing safety standards in healthcare.

## Part II: The Survey Instrument

### Survey on the Use, Cleaning, and Disinfection Management of Medical Radiation Protective Apparel

**Instructions for Respondents:** Thank you for participating in this important survey. Your responses will help us understand and improve the management of medical radiation protective apparel within our healthcare system. Please answer each question to the best of your ability. The survey is divided into seven sections. Please follow the specific instructions provided at the beginning of each section.

### Section I: General Information

**Instructions:** This section collects demographic data about your institution and the departments that utilize medical radiation protective apparel. Please select the most appropriate answer(s) for each question. For questions with an "Other" option, please provide a specific description in the space provided.

**1. What is the classification of your hospital?**

- ☐ Tertiary Grade A
- ☐ Tertiary Grade B
- ☐ Secondary Grade A
- ☐ Secondary Grade B
- ☐ Private Hospital
- ☐ Other (Please specify): ______________________

**2. What is the nature of your hospital?**

- ☐ Comprehensive
- ☐ Specialized

**3. If a specialized hospital, please specify the type:**

- ☐ Oncology Hospital
- ☐ Obstetrics and Gynecology Hospital
- ☐ Traditional Chinese Medicine Hospital
- ☐ Stomatology / Dental Hospital
- ☐ Dermatology Hospital
- ☐ Other (Please specify specialty): ______________________

**4. Which departments in your facility provide medical radiation protective apparel?** [Multiple-Choice]

- ☐ Interventional Catheterization Laboratory (Cath Lab)
- ☐ Operating Room / Surgical Services
- ☐ Department of Nuclear Medicine
- ☐ All of the above
- ☐ Other (Please specify): ______________________

**5. Which departments in your facility have the highest usage of medical radiation protective apparel?** [Multiple-Choice]

- ☐ Cardiology / Cardiac Surgery
- ☐ Neurology / Neurosurgery
- ☐ Interventional Vascular Department
- ☐ Gastroenterology
- ☐ Oncology
- ☐ Radiology
- ☐ Interventional Catheterization Laboratory (Cath Lab)
- ☐ All of the above
- ☐ Other (Please specify): ______________________

**6. What are the most commonly used types of medical radiation protective apparel in your facility?** [Multiple-Choice]

- ☐ Lead Apron (Vest/Skirt style)
- ☐ Lead Cap
- ☐ Lead Thyroid Collar
- ☐ Lead Gloves
- ☐ Leaded Eyewear
- ☐ Lead Protective Skirt (Wrap-around)
- ☐ Lead Vest
- ☐ Lead Trousers/Pants
- ☐ Mobile Lead Shield/Screen
- ☐ All of the above
- ☐ Other (Please specify): ______________________

### Section II: Administrative and Management Oversight of Cleaning and Disinfection

**Instructions:** This section investigates the administrative structure and management model for the cleaning and disinfection of medical radiation protective apparel. Please select the most appropriate answer(s).

**7. Does your hospital have a unified management system for the cleaning and disinfection of medical radiation protective apparel?**

- ○ Yes
- ○ No

**8. To which administrative department does the responsibility for cleaning and disinfection of medical radiation protective apparel belong?** [Multiple-Choice]

- ☐ Nursing Department
- ☐ Infection Prevention and Control (IPC) Department
- ☐ Logistics / Facilities Management Department
- ☐ Other (Please specify): ______________________

**9. What is the management model for the cleaning and disinfection of medical radiation protective apparel in your facility?**

- ☐ Each department manages its own cleaning and disinfection independently.
- ☐ The hospital has a centralized management system for cleaning and disinfection.
- ☐ This issue is not actively monitored or managed.
- ☐ Other (Please specify): ______________________

**10. Which entity is responsible for performing the washing/laundering of medical radiation protective apparel in your facility?** [Multiple-Choice]

- ☐ In-house hospital laundry services
- ☐ Contracted professional third-party laundering service
- ☐ Contracted general housekeeping/janitorial company
- ☐ Personnel within the user department (e.g., nurses, technicians)
- ☐ Other (Please specify): ______________________

**11. Is there a designated person or role specifically responsible for managing the cleaning and disinfection of medical radiation protective apparel?**

- ○ Yes
- ○ No

**12. (If you answered "No" to question 11) What are the reasons for not having a designated person responsible for this task?** [Multiple-Choice]

- ☐ Lack of awareness or attention to the cleaning and disinfection of this apparel.
- ☐ Belief that disinfection is unnecessary for this type of apparel.
- ☐ High workload and lack of personnel to dedicate to this task.
- ☐ Concern that cleaning/disinfection processes may damage the apparel.
- ☐ Other (Please specify): ______________________

**13. Who is primarily responsible for the day-to-day oversight of cleaning and disinfection of medical radiation protective apparel?**

- ☐ Department Head / Chief of Service
- ☐ Nurse Manager / Head Nurse
- ☐ Charge Nurse / Nursing Team Leader
- ☐ Designated Staff Nurse
- ☐ Other (Please specify role): ______________________

**14. Who is primarily responsible for the physical implementation (i.e., performing the work) of cleaning and disinfection of medical radiation protective apparel?**

- ☐ Physicians
- ☐ Nurses
- ☐ Housekeeping / Environmental Services Staff
- ☐ Other (Please specify role): ______________________

### Section III: User and Cleaning/Disinfection Personnel Survey

**Instructions:** This section gathers information about the individuals who use the apparel and those who perform the cleaning and disinfection, including their training. Please select the most appropriate answer(s).

**15. Who are the primary users of medical radiation protective apparel in your facility?** [Multiple-Choice]

- ☐ Physicians
- ☐ Nurses and Technologists
- ☐ Patients
- ☐ Housekeeping / Environmental Services Staff
- ☐ All of the above
- ☐ Other personnel (Please specify): ______________________

**16. Does your facility's management provide pre-service training to healthcare professional users of medical radiation protective apparel?**

- ○ Yes
- ○ No

**17. What topics are covered in the pre-service training for healthcare professional users?** [Multiple-Choice]

- ☐ Proper donning and doffing procedures
- ☐ Precautions and best practices during use
- ☐ Proper storage procedures (e.g., hanging on approved racks)
- ☐ Requirements for storage location and environment
- ☐ Requirements for routine integrity testing and inspection
- ☐ Other (Please specify): ______________________
- ☐ All of the above

**18. Who are the personnel responsible for cleaning and disinfecting medical radiation protective apparel?** [Multiple-Choice]

- ☐ Physicians
- ☐ Nurses
- ☐ Housekeeping / Environmental Services Staff
- ☐ Other (Please specify): ______________________

**19. Does your facility's management provide relevant training for personnel responsible for disinfecting medical radiation protective apparel?**

- ○ Yes
- ○ No

**20. What topics are covered in the training for disinfection personnel?** [Multiple-Choice]

- ☐ Procedures and precautions for cleaning and disinfection
- ☐ Procedures for proper storage of cleaned apparel
- ☐ Procedures for the safe transport of contaminated and cleaned apparel
- ☐ Other (Please specify): ______________________
- ☐ All of the above

**21. Is there a competency assessment and/or certification process following the training for disinfection personnel?**

- ○ Yes
- ○ No

**22. Do disinfection personnel receive periodic training for continuous quality improvement?**

- ○ Yes
- ○ No

### Section IV: Disinfection Equipment and Methods

**Instructions:** This section focuses on the specific equipment and chemical agents used for the cleaning and disinfection of medical radiation protective apparel. Please select all that apply.

**23. What equipment is available in your facility for the cleaning and disinfection of medical radiation protective apparel?** [Multiple-Choice]

- ☐ Ultraviolet (UV) disinfection cabinet
- ☐ Ozone disinfection cabinet
- ☐ Automated spray-washing machine
- ☐ Isolated drying cabinet/machine
- ☐ Specialized disinfection and drying cabinet
- ☐ Integrated cleaning and disinfection cabinet
- ☐ Manual cleaning and disinfection (e.g., hand-wiping)
- ☐ Other (Please specify): ______________________

**24. What methods are used for the rapid cleaning and disinfection of medical radiation protective apparel in your facility?** [Multiple-Choice]

- ☐ Hydrogen peroxide disinfectant wipes
- ☐ Alcohol-based wipes or sprays
- ☐ Chlorine-based (bleach) disinfectant wipes or sprays
- ☐ Quaternary ammonium compound disinfectant wipes or sprays
- ☐ Other (Please specify): ______________________
- ☐ All of the above

**25. Is there a designated storage area specifically for cleaned and disinfected medical radiation protective apparel?**

- ○ Yes
- ○ No

**26. Where is cleaned medical radiation protective apparel typically stored?** [Multiple-Choice]

- ☐ In a dedicated, designated room
- ☐ In a dedicated, designated cabinet or on approved racks
- ☐ No specific location; stored arbitrarily
- ☐ Other (Please specify): ______________________

**27. What is the sorting process for used medical radiation protective apparel?**

- ☐ Sorted immediately after use at the point of care.
- ☐ Collected and transported to a central location for sorting.
- ☐ Other (Please specify): ______________________

**28. Where is contaminated (used) medical radiation protective apparel placed pending cleaning?**

- ☐ In a designated collection area
- ☐ In a dedicated collection bin or cabinet
- ☐ No designated collection area or container
- ☐ Other (Please specify): ______________________

**29. Does your facility maintain a log or record for the cleaning and disinfection of medical radiation protective apparel?**

- ○ Yes
- ○ No

### Section V: Policies and Procedures Survey

**Instructions:** This section assesses the existence and scope of formal written policies and procedures related to medical radiation protective apparel. Please select the most appropriate answer(s).

**30. Does your facility have written policies or standard operating procedures (SOPs) related to medical radiation protective apparel?**

- ○ Yes
- ○ No

**31. Which of the following elements are included in your facility's policies/SOPs for medical radiation protective apparel?** [Multiple-Choice]

- ☐ Policy for designated personnel management and apparel inventory/registration
- ☐ SOPs for workflow and safe handling procedures
- ☐ Protocols for segregation, collection, cleaning, and disinfection
- ☐ Guidelines for storage management and periodic quality control sampling (e.g., microbial testing)
- ☐ Procedures for equipment maintenance and environmental hygiene
- ☐ Defined job responsibilities and occupational safety/PPE requirements for staff
- ☐ Quality control protocols for outsourced transport vehicles
- ☐ Other (Please specify): ______________________
- ☐ All of the above

### Section VI: Environmental and Quality Monitoring Survey

**Instructions:** This section examines the methods used for quality control, environmental monitoring, and traceability of medical radiation protective apparel. Please select the most appropriate answer(s).

**32. For apparel used in a Class 100 laminar flow (ISO Class 5) environment, such as an operating room or cath lab, is there a full-process information tracking and traceability system for its cleaning and disinfection?**

- ○ Yes
- ○ No

**33. What form of traceability management is used for the cleaning and disinfection of medical radiation protective apparel in your facility?**

- ☐ Fully manual record-keeping (e.g., paper logs)
- ☐ Partially manual record-keeping
- ☐ Fully electronic/information system-based traceability
- ☐ Partially electronic/information system-based traceability
- ☐ Other (Please specify): ______________________

**34. How often does your facility conduct quality assurance testing on its medical radiation protective apparel?**

- ☐ Every month
- ☐ Every 3 months
- ☐ Every 6 months
- ☐ Annually (once per year)
- ☐ Less than once per year
- ☐ Never tested
- ☐ Other (Please specify frequency): ______________________

**35. Which department is typically responsible for conducting this quality assurance testing?** [Multiple-Choice]

- ☐ Nursing Department
- ☐ Medical Affairs / Medical Staff Office
- ☐ Infection Prevention and Control (IPC) Department
- ☐ Radiology / Medical Physics Department
- ☐ A higher-level governing body or external service
- ☐ Other (Please specify): ______________________

**36. Is a formal test report issued after the quality assurance testing is completed?**

- ○ Yes
- ○ No

**37. What metrics are included in the periodic quality assurance testing of your medical radiation protective apparel?**

| - Metric | - Please check if this is part of your regular testing protocol |
| --- | --- |
| - Attenuation Properties / Lead (Pb) Equivalency | - ☐ |
| - Microbial Content (Bioburden) | - ☐ |
| - Physical Integrity (e.g., tears, cracks, seam failures) | - ☐ |
| - Other (Please specify): ______________________ | - ☐ |
| - All of the above | - ☐ |

**38. Does your facility conduct periodic environmental monitoring (e.g., surface cultures) of the storage areas for medical radiation protective apparel?**

- ○ Yes
- ○ No

### Section VII: Department-Specific Survey

**Instructions:** The following questions are specific to your department's practices. Please answer based on the standard procedures within your direct working area.

**39. What is your department?**

- ☐ Interventional Catheterization Laboratory (Cath Lab)
- ☐ Operating Room / Surgical Services
- ☐ Department of Nuclear Medicine
- ☐ Radiology Department
- ☐ Stomatology / Dental Department

**40. What are the most commonly used types of medical radiation protective apparel in your department?** [Multiple-Choice]

- ☐ Lead Apron (Vest/Skirt style)
- ☐ Lead Cap
- ☐ Lead Thyroid Collar
- ☐ Lead Gloves
- ☐ Leaded Eyewear
- ☐ Lead Protective Skirt (Wrap-around)
- ☐ Lead Vest
- ☐ Lead Trousers/Pants
- ☐ Other (Please specify): ______________________
- ☐ All of the above

**41. What equipment is available in your department for the cleaning and disinfection of medical radiation protective apparel?** [Multiple-Choice]

- ☐ Ultraviolet (UV) disinfection cabinet
- ☐ Ozone disinfection cabinet
- ☐ Automated spray-washing machine
- ☐ Isolated drying cabinet/machine
- ☐ Specialized disinfection and drying cabinet
- ☐ Integrated cleaning and disinfection cabinet
- ☐ Manual cleaning and disinfection (e.g., hand-wiping)
- ☐ Other (Please specify): ______________________

**42. What methods are used for the cleaning and disinfection of medical radiation protective apparel in your department?** [Multiple-Choice]

- ☐ Hydrogen peroxide disinfectant wipes
- ☐ Alcohol-based wipes or sprays
- ☐ Chlorine-based (bleach) disinfectant wipes or sprays for rapid disinfection
- ☐ Quaternary ammonium compound disinfectant wipes or sprays
- ☐ Other (Please specify): ______________________

**43. What is the frequency of disinfection for medical radiation protective apparel in your department?**

- ☐ Disinfected after every single use ("one person, one use, one disinfection")
- ☐ Disinfected centrally at the end of each day
- ☐ Disinfected centrally at the end of each week
- ☐ This issue is not actively monitored or managed
- ☐ Other (Please specify frequency): ______________________

**44. Where is cleaned and disinfected medical radiation protective apparel stored in your department?**

- ☐ In a dedicated disinfection cabinet
- ☐ In a sterile supply room
- ☐ In a designated clean collection area
- ☐ Other (Please specify): ______________________

**45. In which area is medical radiation protective apparel typically donned (put on) by users in your department?**

- ☐ Sterile area
- ☐ Clean area
- ☐ Semi-clean area
- ☐ Contaminated area
- ☐ Not monitored / No specific area

**46. In which area is medical radiation protective apparel typically doffed (taken off) by users in your department?**

- ☐ Clean area
- ☐ Contaminated area
- ☐ Semi-contaminated area
- ☐ Arbitrarily removed in any location
- ☐ Other (Please specify): ______________________

**47. In the operating room, what is the rapid disinfection method used if apparel becomes contaminated mid-procedure?** [Multiple-Choice]

- ☐ Wiped with a damp cloth
- ☐ Sprayed or wiped with an alcohol-based disinfectant for rapid disinfection
- ☐ Sprayed or wiped with a chlorine-based disinfectant for rapid disinfection
- ☐ Immediate replacement of the protective apparel
- ☐ Immediate replacement of the outer surgical gown
- ☐ Other (Please specify): ______________________

**48. In the operating room, what is the pre-treatment for apparel that has been contaminated with visible soil (e.g., blood, bodily fluids)?**

- ☐ Wiped with a damp cloth
- ☐ Wiped or sprayed with a chlorine-based disinfectant
- ☐ Wiped or sprayed with an alcohol-based disinfectant
- ☐ Wiped or sprayed with a quaternary ammonium compound disinfectant
- ☐ Other (Please specify): ______________________

## Part III Provided Upon Completion of the Questionnaire ：Appendix A: Synopsis of Best Practices for Apparel Lifecycle Management

Upon completion of the questionnaire, we will provide you with a document outlining the evidence-based best practices for the comprehensive management of medical radiation protective apparel, based on the latest and most widely recognized international standards. This guide synthesizes best practices across the key phases of the apparel's lifecycle. Adherence to these practices can help your facility enhance safety, ensure regulatory compliance, and maximize the lifespan of your apparel.

### 1. Cleaning and Disinfection Protocol

Effective hygiene protocols are fundamental to preventing MRPA from becoming a source of cross-contamination. The process must address the challenge of biofilm and use appropriate, non-damaging agents.

- **Frequency:**
  - **Routine Cleaning/Disinfection:** Apparel should be wiped down after every use, between procedures. This is a key recommendation from bodies like AORN and helps manage surface contamination and slow biofilm formation.
  - **Scheduled Deep Cleaning:** A thorough, deep cleaning and sanitization process should be conducted on a regular schedule, with quarterly (every 3 months) being the most widely cited best practice for high-touch items like lead aprons.
- **Method (The Two-Step Process):**
  1. **Step 1: Cleaning with Friction:** The first and most critical step is to physically clean the garment. This involves applying an approved cleaning agent with a soft brush or cloth and using mechanical friction (scrubbing) to break down and remove visible soil, organic matter, and, most importantly, invisible biofilm. Wiping alone is insufficient.
  2. **Step 2: Disinfection:** After the surface has been thoroughly cleaned, an approved disinfectant is applied. The disinfectant must be allowed to remain on the surface for the full contact time specified by the manufacturer to ensure efficacy.
- **Approved and Prohibited Agents:** The choice of chemical agent is critical to avoid damaging the apparel's protective core and outer fabric.

| Agent Category | Recommendation & Rationale | Examples |
| --- | --- | --- |
| **Recommended Agents** |  |  |
| Hydrogen Peroxide-Based Wipes | Effective broad-spectrum disinfectants that are generally compatible with apron materials and do not leave harmful residues. | Clorox Healthcare® Hydrogen Peroxide Wipes |
| Quaternary Ammonium Compounds (QACs) | Effective against a wide range of bacteria and viruses. Alcohol-free, water-based formulations are recommended to prevent material degradation. | Ecolab® Quaternary Based Wipes, Lysol® IC™ Quaternary Disinfectant, Sani-Cloth® AF3 (Grey Top) |
| Mild Soaps/Detergents | Suitable for the initial cleaning step to remove soil and stains. Must be thoroughly rinsed off before disinfection. | Mild dishwashing liquid (e.g., Dawn®), specialized apron cleaners (e.g., Revolution Scrubbles®) |
| **Prohibited Agents** |  |  |
| Bleach (Sodium Hypochlorite) | Highly corrosive and can degrade both the outer fabric and the inner protective materials, compromising the apron's integrity and shortening its lifespan. | Any cleaner containing bleach. |
| Alcohol-Based Solutions | Can dry out, crack, and degrade the vinyl or nylon outer covering of the apron, leading to premature failure and potential damage to the attenuating core material. | Isopropyl alcohol wipes, ethanol-based sprays. |
| Abrasive or Harsh Solvents | Can physically damage the surface of the apron, creating sites for microbial harborage and material breakdown. | Scouring powders, industrial solvents, acetone. |

### 2. Storage and Handling

Proper storage is arguably as important as cleaning for preserving the integrity and lifespan of radiation protective apparel. Improper storage is a leading cause of damage to the internal lead or lead-composite layers.

- **DO:**
  - **Use Approved Hangers/Racks:** Always store apparel on specially designed, robust hangers or racks that support the full weight from the shoulders. This prevents stress on the material.
  - **Store Unobstructed:** Ensure garments hang freely without being compressed or bent.
  - **Secure Fasteners:** Close any hook-and-loop (Velcro) or buckle closures before hanging to prevent snagging and tearing.
- **DO NOT:**
  - **Fold or Crease:** Never fold, crease, or sharply bend an apron. This is the most common cause of cracks in the internal protective material, which renders the apron unsafe.
  - **Drape Over Edges:** Do not drape apparel over chairs, equipment, or sharp-edged surfaces.
  - **Pile or Stack:** Do not pile garments on top of one another on a shelf or on the floor. Weight will cause creasing and damage.

### 3. Inspection and Integrity Testing

Regular inspection is a regulatory requirement and an essential practice to ensure the apparel continues to provide adequate protection.

- **Routine Inspection:** Users should perform a quick visual and tactile inspection before each use, checking for obvious signs of damage such as tears, punctures, exposed core material, or stiff, cracked areas.
- **Annual Formal Inspection:** A comprehensive inspection must be performed at least annually, as required by The Joint Commission and other regulatory bodies. This inspection should include:
  - **Visual and Tactile Check:** A thorough examination for any signs of wear and tear.
  - **Radiographic/Fluoroscopic Examination:** Using X-ray imaging to inspect the internal core material for cracks, holes, or thinning that are not visible to the naked eye. This is the definitive test of the apron's integrity.
- **Documentation:** All inspections, findings, and actions (e.g., repair, disposal) must be meticulously documented for each individual garment. This creates an auditable record essential for compliance.
- **Rejection Criteria:** A formal policy should define when a garment must be removed from service. Established criteria include defects larger than a specified area, particularly over critical organs :
  - Defects > 15 mm^2^ over the thyroid, chest, or gonad regions.
  - Defects > 11 mm^2^ on a thyroid shield.
  - Defects > 670 mm^2^ on seams or overlapped areas.

### 4. Personnel Training and Accountability

Technology and policies are only effective if personnel are trained and held accountable for their implementation.

- **Training Content:** All personnel who use or handle MRPA should receive training that covers the full lifecycle: proper donning/doffing, correct use, cleaning and disinfection procedures, proper storage and handling, how to perform routine inspections, and when to report damage.
- **Establishing Accountability:** Facilities should have clear, written policies that assign specific responsibility for cleaning, inspection, and management. This closes the "accountability gap" where critical tasks are neglected because of an "it's not my job" mentality. This may involve designating a lead person in each department or creating a centralized management role.

### 5. Disposal

When an apron reaches the end of its service life or fails inspection, it must be disposed of correctly.

- **Lead-Containing Apparel:** Garments containing lead are considered hazardous waste. They cannot be disposed of in regular trash and must be handled by a certified hazardous waste carrier or a specialized disposal service.
- **Lead-Free Apparel:** Garments made from non-hazardous composite materials (e.g., bismuth, tin) can typically be disposed of as standard waste, though institutional and local regulations should always be confirmed.
