# Supplementary material for: Organizational and Nursing Management Factors Associated With Guideline‐Discordant Medical Radiation Protective Apparel Disinfection: A Multicenter Interpretable Machine Learning Study
Source: J Nurs Manag. 2026 Nov 8;2026:2036458. doi: 10.1155/jonm/2036458 (PMC13745874; doi:10.1155/jonm/2036458)
Supplement: Supplementary file 3 [file JONM-2026-2036458-s009.docx]

**Supplementary2: Mapping of Survey Items to Machine Learning Features**

This table describes how raw survey responses from the "Survey on the Management of Medical Radiation Protective Apparel" were operationalized and encoded for the machine learning models.

| **Feature Category** | **Model Variable Name** | **Source Question (Q#)** | **Encoding & Scoring Logic** |
| --- | --- | --- | --- |
| **Institutional Context** | Hosp_Grade_3A | Q1 | Binary: 1 = Tertiary A; 0 = Other |
|  | Hosp_Grade_3B | Q1 | Binary: 1 = Tertiary B; 0 = Other (Ref: Secondary A) |
| **Governance Structure** | Unified_Mgmt_for_ MRPA | Q7 | Binary: 1 = Yes; 0 = No |
|  | MRPA_Management_by_Hospital | Q9 | Binary: 1 = Centralized Hospital Mgmt; 0 = Other |
|  | MRPA_Management_by_Dept | Q9 | Binary: 1 = Department-level Mgmt; 0 = Other |
| **Personnel Roles** | MRPA_Has_Designated_Personnel | Q11 | Binary: 1 = Yes; 0 = No |
|  | MRPA_Manager_is_Head_nurse | Q13 | Binary: 1 = Head Nurse; 0 = Other |
|  | MRPA_Manager_is_Dept_Head | Q13 | Binary: 1 = Department Head; 0 = Other |
|  | MRPA_Manager_is_Dedicated_Nurse | Q13 | Binary: 1 = Dedicated/Specialized Nurse; 0 = Other |
|  | MRPA_Implementer_is_Nurse | Q14 | Binary: 1 = Registered Nurse; 0 = Other |
|  | MRPA_Implementer_is_Doctor | Q14 | Binary: 1 = Physician; 0 = Other |
|  | MRPA_Implementer_is_Cleaner | Q14 | Binary: 1 = Cleaning Staff; 0 = Other |
| **Training & Policy** | MRPA_Users_are_Trained | Q16 | Binary: 1 = Pre-service training provided; 0 = No |
|  | MRPA_Disinfectors_are_Trained | Q19 | Binary: 1 = Specific disinfection training; 0 = No |
|  | Has_Policy_Usage | Q30/31 | Binary: 1 = Clear usage/disinfection policy; 0 = No |
|  | Has_CQI_Training | Q22 | Binary: 1 = Continuous Quality Improvement training; 0 = No |
| **Workflow & Systems** | Has_Designated_Storage_for_Clean_MRPA | Q25 | Binary: 1 = Dedicated clean storage; 0 = Other |
|  | Has_Traceability_for_Critical_Area | Q33 | Binary: 1 = Digital or QR-based tracking; 0 = None/Manual |
|  | Has_End_to_End_Digital_Traceability | Q33 | Binary: 1 = Integrated digital tracking; 0 = Other |
|  | Washing_Dept_InHouse | Q10 | Binary: 1 = Internal CSSD/Laundry; 0 = Other |
|  | Washing_Dept_ThirdParty | Q10 | Binary: 1 = Outsourced Third-party; 0 = Other |
|  | Washing_Dept_DeptItself | Q10 | Binary: 1 = Cleaned within clinical dept; 0 = Other |
| **Target Variable** | **is_CNP** | **Q24** | **1 = Uses prohibited Alcohol/Chlorine (Yes_CNP); 0 = Uses neutral detergents/validated wipes (No_CNP)** |

**Note:** "Other" in binary encoding refers to any response category not explicitly listed as 1. All variables were checked for multicollinearity before model inclusion.
